# Surgical Training During The COVID-19 Pandemic

**DOI:** 10.12669/pjms.37.2.4119

**Published:** 2021

**Affiliations:** 1 Rupali Shah, MBBCh Surgical Simulation Fellow, London. UK; 2 Isabelle Carty, MBBS. Medical Education Fellow, Dartford. UK; 3 Francis Ugwu, MBBS, MRCS. Surgical Simulation Fellow, London. UK

Due to the lack of surgical training opportunities both during the COVID pandemic and to some extent prior to it, this detailed laparoscopic curriculum would be beneficial for surgical trainees to enhance their technical skills in a safe environment prior to undertaking procedures on patients.

The Covid-19 pandemic has had a transformative effect on surgical training in the United Kingdom that has been predominantly negative.^1^,^2^ A number of causative points have contributed to this and these include; the decision to minimise personnel in the operating theatres (meaning that operations and care were more senior led), the cancellation of elective procedures to protect “well” patients from unnecessary exposure to the virus, the redeployment of surgical trainees to ITU and medical wards as well as changes in shift schedules.^2^

A survey was conducted by the authors and circulated among the junior trainees (all grades below speciality trainee registrars) in the general surgery department to identify key concerns with regards to surgical training. The results were unanimously conclusive of a lack of training opportunities in theatres.

In order to satisfy this deficit in training, the authors devised a surgical teaching curriculum whereby trainees would be allocated time in the simulation room which was equipped with laparoscopic box trainers as well as virtual reality laparoscopic trainers with haptic feedback. Simulators provide a safe method of training without compromising patient safety.^3^,^4^

The curriculum included simple tasks for the trainees to practice with increasing levels of complexity. These tasks included the transferral of cotton wool balls, transferral of black eyed beans, stacking sugar cubes, placing polo mints onto a string, peg board transfer, cutting a circle in glove, intracorporeal knot tying, intracorporeal clip application, appendicectomy and cholecystectomy. Additionally, we created an individualised online database for trainees to record their progress, including simulation modality, type of supervision, time taken, complications as well as total time spent in the simulation laboratory.

Each of the skills practiced and mastered would enable the trainee to carry out a more complex task. For example, starting off with the cotton wool ball transfer enabled the trainees to acquaint themselves with the spatial awareness and depth perception required to navigate the instruments in order to adequately perform the task. They would then move on to transferring smaller black eyed beans, which would require more precision. Whilst stacking the sugar cubes may initially appear quite simple to trainees, it is vital in building their manual dexterity skills and ensuring that they have become more precise with the placement of the instruments and materials. This is reinforced during the peg board transfer. Cutting a circle in a glove highlights the importance of sharp safety, especially intra-abdominally. It also encourages trainees to make smoother movements to avoid ragged edges on the cut they have made in the glove. Intracorporeal clip application and knot tying further build on this precision.

Once all the basic laparoscopic skills had been mastered, the trainees were then able to practice appendicectomies and cholecystectomies on the virtual reality laparoscopic simulators. The feedback from trainees after practicing with the virtual reality kits was overwhelmingly positive. They felt that the haptic feedback made the experience more realistic. Additionally, the software allowed each trainee to track their progress by timing the procedures, measuring blood loss and assessing their economy of movement.

All skills featured in these simulated sessions are transferrable and feedback from trainees that participated have shown increased levels of coordination and confidence when operating in actual theatres.^3^ Therefore, this provides a very good argument for the integration of simulation into surgical training in the United Kingdom in order to both improve hand-eye coordination, supplement exposure and experience to more common cases as well as boosting the confidence of trainees.^4^,^5^
